# A Large-Scale Community-Based Outbreak of Paratyphoid Fever Caused by Hospital-Derived Transmission in Southern China

**DOI:** 10.1371/journal.pntd.0003859

**Published:** 2015-07-17

**Authors:** Meiying Yan, Bo Yang, Zhigang Wang, Shukun Wang, Xiaohe Zhang, Yanhua Zhou, Bo Pang, Baowei Diao, Rusong Yang, Shuyu Wu, John D. Klena, Biao Kan

**Affiliations:** 1 State Key Laboratory for Infectious Disease Prevention and Control, National Institute for Communicable Disease Control and Prevention, Chinese Center for Disease Control and Prevention, Beijing, China; 2 Collaborative Innovation Center for Diagnosis and Treatment of Infectious Diseases, Hangzhou, Zhejiang, China; 3 Center for Disease Control and Prevention of Yuanjiang County, Yunnan, China; 4 Center for Disease Control and Prevention of Yuxi City, Yunnan, China; 5 International Emerging Infections Program, US Centers for Disease Control and Prevention, Beijing, China; 6 Global Disease Detection Branch, Division of Global Health Protection, Center for Global Health, Centers for Disease Control and Prevention, Atlanta, Georgia, United States of America; Massachusetts General Hospital, UNITED STATES

## Abstract

**Background:**

Since the 1990s, paratyphoid fever caused by *Salmonella* Paratyphi A has emerged in Southeast Asia and China. In 2010, a large-scale outbreak involving 601 cases of paratyphoid fever occurred in the whole of Yuanjiang county in China. Epidemiological and laboratory investigations were conducted to determine the etiology, source and transmission factors of the outbreak.

**Methodology/Principal Findings:**

A case-control study was performed to identify the risk factors for this paratyphoid outbreak. Cases were identified as patients with blood culture–confirmed *S*. Paratyphi A infection. Controls were healthy persons without fever within the past month and matched to cases by age, gender and geography. Pulsed-field gel electrophoresis and whole-genome sequencing of the *S*. Paratyphi A strains isolated from patients and environmental sources were performed to facilitate transmission analysis and source tracking. We found that farmers and young adults were the populations mainly affected in this outbreak, and the consumption of raw vegetables was the main risk factor associated with paratyphoid fever. Molecular subtyping and genome sequencing of *S*. Paratyphi A isolates recovered from improperly disinfected hospital wastewater showed indistinguishable patterns matching most of the isolates from the cases. An investigation showed that hospital wastewater mixed with surface water was used for crop irrigation, promoting a cycle of contamination. After prohibition of the planting of vegetables in contaminated fields and the thorough disinfection of hospital wastewater, the outbreak subsided. Further analysis of the isolates indicated that the origin of the outbreak was most likely from patients outside Yuanjiang county.

**Conclusions:**

This outbreak is an example of the combined effect of social behaviors, prevailing ecological conditions and improper disinfection of hospital wastewater on facilitating a sustained epidemic of paratyphoid fever. This study underscores the critical need for strict treatment measures of hospital wastewater and the maintenance of independent agricultural irrigation systems in rural areas.

## Author Summary

Typhoid and paratyphoid fever remain public health concerns for developing countries. From May 2010 to June 2011, a large-scale outbreak involving 601 cases of paratyphoid fever occurred in China. Epidemiological and laboratory investigations were conducted to determine the etiology, source and transmission factors of the outbreak. Farmers and young adults were the populations mainly affected in this outbreak, and the consumption of raw vegetables was the main risk factor associated with paratyphoid fever. We found that hospital wastewater mixed with surface water was used for vegetable irrigation. The contaminated water from hospitals combined with the regional habit of eating uncooked vegetables lead to the massive outbreak of paratyphoid. After prohibition of the planting of vegetables in contaminated fields and the thorough disinfection of hospital wastewater, the outbreak subsided. Molecular subtyping and whole-genome sequencing of *S*. Paratyphi A isolates recovered from improperly disinfected hospital wastewater showed indistinguishable patterns matching most of the isolates from the cases. Further analysis of the isolates indicated that the origin of the outbreak was most likely from patients outside Yuanjiang county. This study underscores the critical need for strict treatment measures of hospital wastewater and the maintenance of independent agricultural irrigation systems in rural areas.

## Introduction

Typhoid and paratyphoid (enteric) fever caused by *Salmonella enterica* serovar Typhi and Paratyphi A remain significant public health problems for developing countries. Globally, 13.5 million cases are estimated to occur annually and are associated with 190,000 deaths in 2010 [1]. Before the 1990s, typhoid cases were more prevalent than paratyphoid cases in Southeast Asia; however, the latter have been steadily increasing [2]. Paratyphoid fever has also been increasingly reported in China since 1998 and has resulted in localized outbreaks in some provinces [3, 4].

Understanding the risk factors of enteric fever is important for prevention and control and to provide tools for interrupting disease transmission in the early phases of an outbreak. In low-prevalence areas, travel and immigration from endemic areas are major risk factors [5]. In high-prevalence countries, the main risk factors include the consumption of unsafe drinking water and contaminated foods and close contact with active cases or carriers [6, 7]. In China, the risk factors for enteric fever differ between urban and rural areas, as well as between coastal cities and inland regions [8, 9].

During epidemic investigations, pulsed-field gel electrophoresis (PFGE) is quite useful for identifying outbreak-associated isolates and source tracing [10]. Recently, whole-genome sequencing has provided increased sensitivity for microbial evolution and molecular epidemiology studies, improving the understanding of disease transmission [11–14].

From May 2010 to June 2011, an outbreak causing 601 cases of paratyphoid fever was documented in Yuanjiang county, Yunnan Province, China. In this study, we report a risk factor analysis coupled with the laboratory-based characterization of outbreak-associated isolates, with the goal of determining the source of the outbreak, implementing control measures and assessing their effectiveness.

## Materials and Methods

### Ethics statement

This study was reviewed and approved by the ethics committee of National Institute for Communicable Disease Control and Prevention, China CDC, according to the medical research regulations of the Ministry of Health, China (ICDC-2014008).

### Epidemiological investigation

A suspect enteric fever case was defined as persistent fever (≥ 37.5°C for more than three days) accompanied by headache and body ache, without obvious upper respiratory or urinary tract infections, trauma or other diagnosed causes of fever. Both suspect cases and laboratory-confirmed cases (culture positive) were reported daily to the Chinese Center for Disease Control and Prevention through an internet-based disease reporting system [15]. Other data were extracted from patient records (e.g., age, sex, home address, work place, occupation, date of presentation to the hospital, suspected or laboratory-confirmed diagnosis). A case-control study with 1:1 matching of controls to cases was initiated and included blood culture–confirmed cases of *S*. Paratyphi A infection and healthy persons without fever during the 1 month prior to the study. The cases and controls were matched for age (no more than 5 years between cases and controls), gender and residential location. Controls were enrolled from households next-door to the households of case subjects. If the case subject lived in a bungalow or stand-alone house, the household to the right of the “case-household” was approached by an epidemiological expert within 1 week of enrollment of the case subject. If the person in that house refused to join the study or failed to meet the enrollment criteria (same gender, within 5 years of the age of the case subject and no fever in 1 month prior to the administration of the questionnaire), the household to the left of the “case-household” was approached, followed by the house parallel to the “case-household” across the street. If the case subject lived in a multistory building, the household to the right of the “case-household” on the same floor was approached. If persons in that house refused to join the study or failed to meet the enrollment criteria, the household to the left of the “case-household” was approached, followed by the household one story above or below the “case-household”. Standard questionnaires administered by local epidemiological experts through face-to-face interview to gather information on a four-week time window of exposure history, including close contact with *S*. Paratyphi A cases, recent history of travel and food and water consumption for all cases and controls. All controls were asked about the time period that coincided with period of exposure history of the matched case subject. All data entry and analyses were performed using SPSS 17.1 for Windows (SPSS Inc., Chicago, IL, USA). The odds ratios (ORs) and exact 95% confidence intervals (CIs) were calculated via a matched multivariate analysis through conditional logistic regression to examine relationships between exposures and illness. To avoid confounding effects, the data of 60% subjects who drank both bottled and municipal water was discarded from the analysis of categorical variables to prevent statistical bias. Statistical significance was designated as a *p* value <0.05.

### Environmental and food samples

Environmental samples of untreated surface water, treated township water, drinking water, hospital waste and suspected food and vegetables were collected and cultured for bacteria. Standard methods for bacterial isolation and identification were utilized [16].

### Clinical laboratory detection

During the outbreak period, samples of blood, stool and urine were collected for the suspect cases. An 8-mL sample of whole blood was injected into a single Bactec culture bottle (Biomerieux, Durham, NC, USA) and incubated at 37°C. Approximately 3 g stool from each patient was used to inoculate selenite broth for enrichment. Urine was centrifuged, and the pellets were suspended in selenite broth and incubated overnight at 37°C. To confirm *S*. Paratyphi A, a serological agglutination reaction was performed using *Salmonella* antisera (S&A Reagents Lab, Bangkok, Thailand) and biochemical tests (Microbact, Medvet Diagnostics, Adelaide, Australia). Antimicrobial susceptibility testing was performed following the Clinical Laboratory Standards Institute (CLSI) broth microdilution method [17]. Antimicrobial agents including ampicillin, amoxicillin/clavulanic acid, ceftriaxone, cefotaxime, ceftazidime, nalidixic acid, ciprofloxacin, chloramphenicol, gentamicin, kanamycin, streptomycin, sulfisoxazole, tetracycline, azithromycin and trimethoprim/sulfamethoxazole were evaluated using CLSI minimum inhibitory concentration (MIC) interpretive standards for Enterobacteriaceae to calculate resistance thresholds [18].

### PFGE

PFGE was performed according to the modified PulseNet protocol for *S*. Paratyphi A [19] using a CHEF DRIII system (Bio-Rad, Hercules, CA, USA). Band similarities were analyzed using BioNumerics software (version 2.5, Applied Maths, Kortrjk, Belgium) by the unweighted pair-group method with arithmetic means method to produce a dendrogram with 1.5% position tolerance. The outbreak strains from Yuanjiang county and the epidemic isolates from Yuxi city were conducted by PFGE analysis and molecular comparison.

### Whole-genome sequencing of *S*. Paratyphi A isolates

Genomic DNA was prepared from overnight cultures using a Wizard Genomic DNA Purification Kit (Promega, Madison, WI, USA) according to the manufacturer’s instructions. Whole-genome sequencing (WGS) was performed on 17 *S*. Paratyphi A isolates using an Illumina HiSeq 2000 with 500-bp paired-end libraries in 8-fold multiplexes (Beijing Genome Institute, Shenzhen, China). The sample set represents geographically dispersed isolates collected over an array of time points (14 months from Feb, 2010 to April, 2011) and multiple PFGE pattern combinations, including 12 outbreak isolates from patients distributed in Yuanjiang county, 2 isolates from wastewater, one contemporaneous isolate from other county, and 2 isolates circulating in Hongta district of Yuxi representing the top 2 PFGE patterns of paratyphoid A. Details of the genomic sequencing of each isolate are summarized in Supplementary S1 Table, including the accession codes of the reads in European Nucleotide Archive (www.ebi.ac.uk/ena).

### *De novo* assembly

The WGS reads were assembled *de novo* using SOAPdenovo2 [20] with optimal K-mer and minimal coverage parameters. Intra-scaffold gaps were filled using GapCloser v1.12 (SOAP package). SOAPaligner v2.21 [21] was used to remap the reads to the assembled scaffolds for validation of the quality of each base that was called in the assembly. The sites were filtered with a quality score of < 20 or a read coverage of < 10 with Bowtie and SAMtools [22].

### SNP calls and phylogenetic inference

A core genome of 4,585,284 bp was obtained by comparing the 17 *S*. Paratyphi A genomes from this study with 6 previously sequenced genomes (S1 Table). The non-repetitive core genome was found to contain 513 SNPs. We identified 270 SNPs after removing SNPs in recombinant regions, as suggested by ClonalFrame [23]. A maximum likelihood tree was inferred with these 270 SNPs in MEGA v5 [24]. The genomic sequence of ATCC9150 was determined as the root because it was most distantly related to the outbreak isolates.

### Accession numbers

All sequence are available at http://www.ebi.ac.uk/ena/data/view/PRJEB9577 and refer to [25, 26]. The accession numbers are: PA1477, ERS747619 SAMEA3451357 PA1477; PA1478, ERS747620 SAMEA3451358 PA1478; PA1815, ERS747621 SAMEA3451359 PA1815; PA1822, ERS747622 SAMEA3451360 PA1822; PA1850, ERS747623 SAMEA3451361 PA1850; PA1886, ERS747624 SAMEA3451362 PA1886; PA1909, ERS747625 SAMEA3451363 PA1909; PA2183, ERS747626 SAMEA3451364 PA2183; PA2184, ERS747627 SAMEA3451365 PA2184; PA2199, ERS747628 SAMEA3451366 PA2199; PA2207, ERS747629 SAMEA3451367 PA2207; PA2216, ERS747630 SAMEA3451368 PA2216; PA2243, ERS747631 SAMEA3451369 PA2243; PA2635, ERS747632 SAMEA3451370 PA2635; PA2641, ERS747633 SAMEA3451371 PA2641; PA2161, ERS747634 SAMEA3451372 PA2161; PA2191, ERS747635 SAMEA3451373 PA2191.

## Results

### Paratyphoid fever epidemic in Yuanjiang

**Fig 1:**
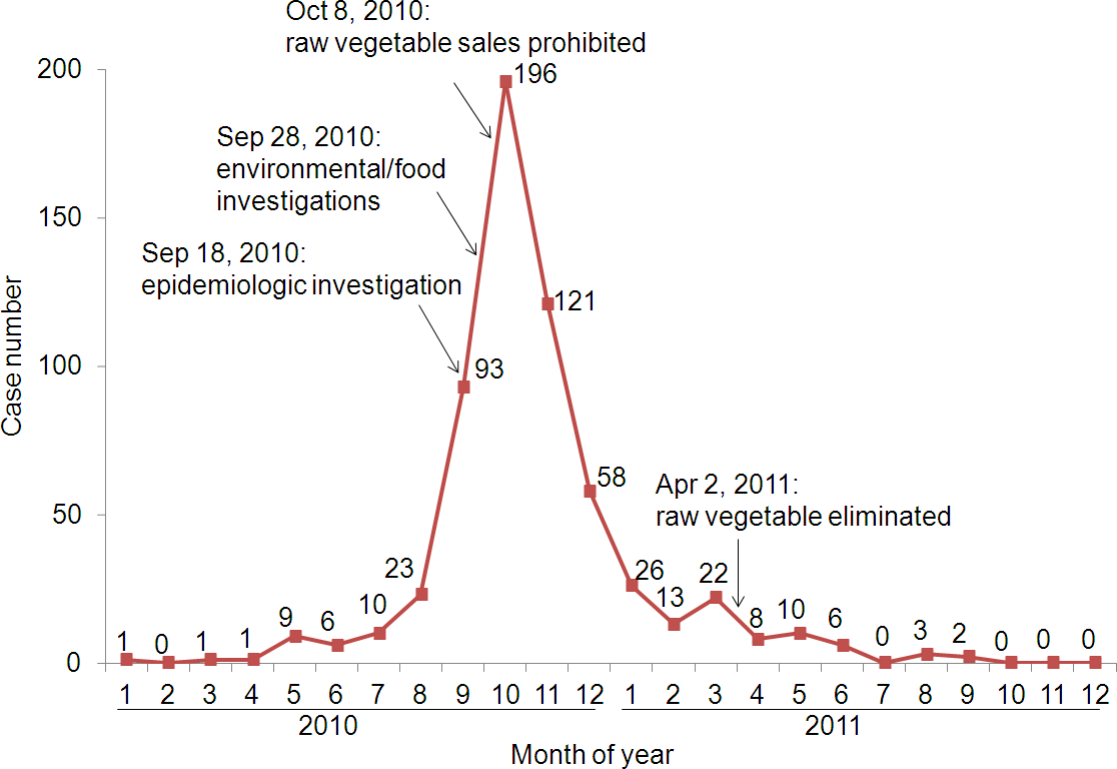
Paratyphoid fever cases reported by month during the outbreak in Yuanjiang. The times of the epidemiological investigations and two interventions are marked with arrows.

From May 2010, a sharp increase in paratyphoid fever A cases was noted in Yuanjiang county (Figs 1 and S1). In August, 23 cases were reported, suggesting that an outbreak of paratyphoid fever was in progress. By the end of 2010, 519 cases of enteric fever were reported (Fig 1); 503 were lab-confirmed via the isolation of *S*. Paratyphi A from blood culture. Since May 2011, no more than 10 cases per month have been reported (Fig 1); only 13 cases were reported in 2012, and three cases in 2013.

Based on the case reporting, the outbreak caused a total of 601 cases from May 2010 to June 2011, involving 10 towns in the county (S2 Fig); most cases (347) were from Lijiang. The incidence in Lijiang reached 708.4/100,000 compared with the average incidence of 45.4/100,000 (1,004 cases) in the Yuxi region and 4.75/100,000 (2,171 cases) in Yunnan Province in 2010. The ratio of male to female cases was 1.3 (1.3:1), and most cases (81%) were in the 20 to 49 [year] age group. Most cases, nearly 61%, were farmers (7.2% (14532/201857) of total population is made up of farmers).

A random sample of 106 paratyphoid cases was reviewed. All patients had fever; other prominently reported signs and symptoms included headache (84%), chills (74%), fatigue (43%), body aches (32%), cough (26%) and dizziness (19%). Most patients presented to the local hospital after one day of fever; 98 cases had a body temperature >37.5°C but less than 39°C, and eight patients had a fever >40°C. The mean fever duration of the cases was three days (range 1–15 days). All *S*. Paratyphi A isolates had the same antibiotic susceptibility testing profile: they were sensitive to cephalosporins and azithromycin (MIC = 8–16 mg/mL) but resistant to nalidixic acid and had decreased susceptibility to ciprofloxacin (MIC = 0.5 mg/mL). Ninety percent of the hospitalized patients were given fluoroquinolones, including oral norfloxacin (0.2 g, three times per day) or intravenous levofloxacin (0.2 g, once a day) combined with ceftriaxone (2.5 g, intravenously, twice per day) for two weeks.

### Epidemiological investigation of disease transmission and associated risk factors

A case-control study was conducted that included 109 pairs of laboratory-confirmed cases and controls matched by age, gender and residential location. As the number of enrolled culture–confirmed cases of paratyphoid fever was more than 100, we chosen the simple 1:1 matching model to conduct the risk association analysis routinely. Eighty (73.40%) cases and their matched controls lived in urban districts of Lijiang town, and the remainder resided in its surrounding areas. Using a multivariate conditional logistic regression analysis, the consumption of raw vegetables (OR = 65.3, 95% CI: 8.3–511.6, P<0.001, Table 1), especially the consumption of raw vegetables not prepared at home (OR = 6.1, 95% CI: 3.1–11.9, P<0.001), was significantly associated with paratyphoid fever. The direct consumption of bottled water (approximately 30% in either group only drank bottled water (unboiled water)) was not associated with the disease (OR = 2.2, P = 0.18). Only 2 cases and 1 control admitted to drinking unboiled municipal water, and this consumption was not shown to have an effect on disease exposure. In addition, no fecal or thermotolerant coliforms were detected in the municipal water of Lijiang. The rates of consumption of uncooked vegetables purchased from Ximen Farm Product Market, small restaurants, supermarkets, canteens and street stalls in cases and controls were also analyzed through conditional logistic regression analysis. The results showed a significant association between paratyphoid fever and the consumption of uncooked vegetables obtained from street stalls (OR = 6.4, 95% CI: 1.9–21.6, P = 0.003), the Ximen Farm Product Market (OR = 18.3, 95% CI: 3.6–93.0, P<0.001), and small restaurants (OR = 29.6, 95% CI: 6.9–127.1, P<0.001).

**Table 1:** Multivariable analysis of potential risk factors for 109 pairs of cases and age- and neighborhood-matched controls during paratyphoid fever outbreak.

Variable	Case exposure (%(n))	Control exposure (%(n))	OR	95% CI	P value
Age(mean(range))	36.9(15–68)	37.2(14–70)	-	-	-
Male sex	53.2(58)	53.2(58)	-	-	-
Drinking water^a^	31.2(34)	26.6(29)	2.2	0.7–7.3	0.18
Consumption of raw vegetables	100(109)	44.0(48)	65.3	8.3–511.6	<0.001
Raw vegetables purchased outside the home	78.0(85)	31.2(34)	6.1	3.1–11.9	<0.001
Raw vegetables made at home	22.0(24)	12.8(14)	2.0	0.9–4.3	0.07
Source of raw vegetables					
Street stall	15.6(17)	4.6(5)	6.4	1.9–21.6	0.003
Supermarket	2.8(3)	4.6(5)	0.9	0.2–4.4	0.93
Ximen Farm Market	16.5(18)	3.7(4)	18.3	3.6–93.0	<0.001
Restaurant	33.9(37)	9.2(10)	29.6	6.9–127.1	<0.001
Canteen	9.2(10)	9.2(10)	2.3	0.6–9.0	0.24

^a^ Only drink bottled water (unboiled water).

**Fig 2:**
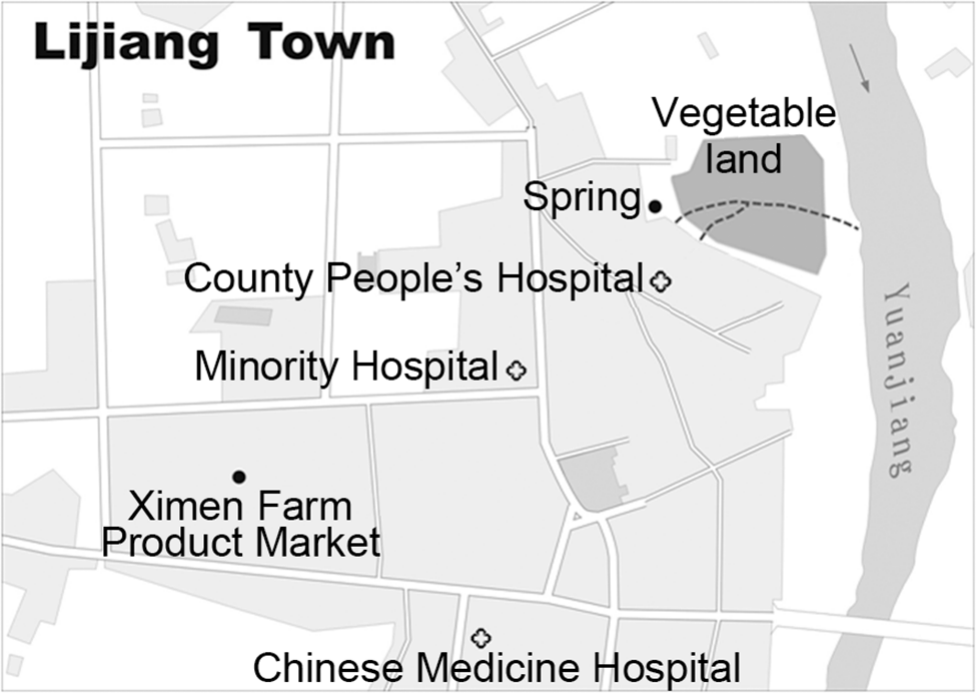
Map of Lijiang town of Yuanjiang county. The vegetable field used for growing raw vegetables near County People’s Hospital is shown as a dark-gray area. The short dashed lines indicate the wastewater from the city and hospital passing through the vegetable field.

Raw vegetables are a favorite food of the local people. Ximen Farm Product Market is the largest vegetable market and supplies farm products for the entire town of Lijiang (Yuanjiang county). The primary source of the cold-served vegetables served at stalls in Ximen Farm Product Market was a vegetable patch located on the east side of Lijiang (Fig 2); this location is also close to County People's Hospital. A gutter from the urban drainage crosses the vegetable patch (Fig 2). Additionally, an open gutter for wastewater discharged directly from the hospital also crossed the land used for growing vegetables, connecting to the urban gutter (Fig 2). The vegetables cultivated in this patch included coriander, mint, green onions and lettuce, all of which were used for the preparation of the cold dishes served in the market. Prior to 2010, farmers used water from a spring near the vegetable patch (Fig 2) to irrigate the vegetables. However, from 2009 to 2010 a severe drought affected most of Yunnan Province, including Yuanjiang. The spring dried up, and beginning in May 2010, the farmers used the wastewater to irrigate vegetables. As mentioned above, wastewater from County People's Hospital, where most enteric fever cases were treated, with 6 isolates recovered from the hospital’s wastewater, was also mixed with the surface water used to cultivate the vegetables, creating a potential for an on-going cycle of contamination.

### Laboratory investigation

**Fig 3:**
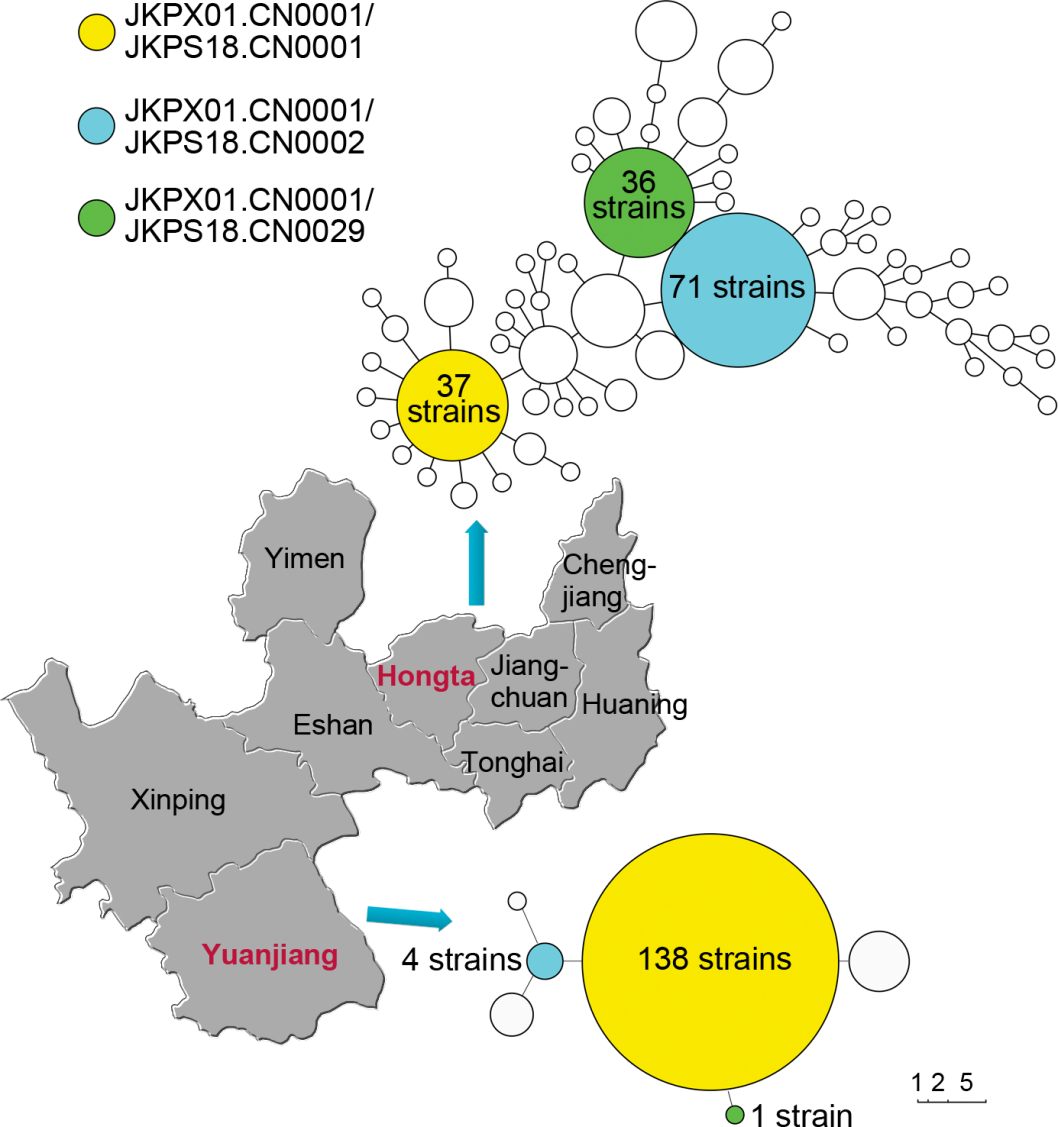
PFGE clusters of *S*. Paratyphi A isolates digested with *Xba*I and *Spe* I. The upper minispanning tree represents the patterns of the Yuxi isolates recovered from 2008 to 2009. The lower tree shows the patterns of the Yuanjiang outbreak isolates in 2010 and 2011. The matched patterns between the Yuanjiang outbreak isolates and the Yuxi endemic isolates are marked in yellow, blue and green.

In total, 630 *S*. Paratyphi A isolates were obtained from the blood, stool and urine of patients during the outbreak. In total, 160 isolates recovered at different times and places during the outbreak, including 10 isolates recovered from wastewater, were analyzed by PFGE. Seven unique strain patterns were identified; 138 isolates, including the 10 from the sewage samples, clustered with the dominant pattern JKPX01.CN0001/JKPS18.CN0001 (Figs 3 and S3). The PFGE pattern similarity for 97% isolates was greater than 98% and included three distinct but similar patterns: JKPX01.CN0001/JKPS18.CN0001, JKPX01.CN0001/JKPS18.CN0109 and JKPX01.CN0001/JKPS18.CN0003. In addition, the dominant pattern, JKPX01.CN0001/JKPS18.CN0001, was indistinguishable from that attributed to *S*. Paratyphi A isolates from the city of Yuxi during the period of 2008 to 2009 (Figs 3 and S3). These data suggest that the outbreak in Yuanjiang may have originated from a previous outbreak in Yuxi.

Laboratory testing of 13 vegetable samples from Ximen Farm Product Market failed to yield any *S*. Paratyphi A; however, abundant fecal coliforms (≥24,000 MPN/100 g) were recovered, well in excess of the limit of 100 MPN/100 g according to the standard of Microbiological Examination of Food hygiene in China. High coliform counts were detected from mint and lettuce samples, demonstrating fecal contamination of these vegetables.

In October and November 2010, 6 isolates of *S*. Paratyphi A were recovered from 10 wastewater outfall samples obtained from County People’s Hospital (three different collection times). Additionally, four *S*. Paratyphi A isolates were recovered from eight wastewater samples collected from two additional hospitals, Minority Hospital and Chinese Medicine Hospital, both of which had treated enteric fever cases. The wastewater from these hospitals was not treated, and both discharged their wastewater directly into the covered urban drainage system (Fig 2). No *S*. Paratyphi A was detected from the effluent of the three main urban sewage outfalls of the county. Nonetheless, the amount of fecal coliforms (≥16,000 MPN/L) exceeded the threshold of 10,000 MPN/L according to the discharge standards for urban sewage treatment. PGFE was performed on the ten isolates from the wastewater, and only one pattern combination (JKPX01.CN0001/JKPS18.CN0001) was obtained, which was indistinguishable from the predominant pattern of isolates from the patients of the Yuanjiang epidemic (Figs 3 and S3).

### Molecular comparison of Yuanjiang outbreak and Yuxi epidemic isolates

The *S*. Paratyphi A isolates (n = 286) recovered from patients living in Hongta district in 2008 and 2009 were analyzed by PFGE. Although a diversity of *Xba*I and *Spe*I pattern combinations were found, one dominant pattern, JKPX01.CN0001/JKPS18.CN0001, was indistinguishable from the dominant pattern of the Yuanjiang outbreak. Other minor PFGE pattern combinations found in the Yuanjiang outbreak, including JKPX01.CN0001/JKPS18.CN0002 and JKPX01.CN0001/JKPS18.CN0029, were also present as common patterns in the Hongta district epidemic. Two patients that represent probable index cases in the Yuanjiang outbreak worked in the Hongta district and had returned to Yuanjiang county for the treatment of enteric fever in Yuanjiang County People's Hospital, providing a potential mechanism of paratyphoidal fever transmission from the Yuxi urban area. The combinations JKPX01.CN0001/JKPS18.CN0003, JKPX01.CN0001/JKPS18.CN0109 and JKPX01.CN0001/JKPS18.CN0110 were not found in the Hongta district epidemic isolates, suggesting the possible mutation of some isolates during the outbreak in Yuanjiang.

**Fig 4:**
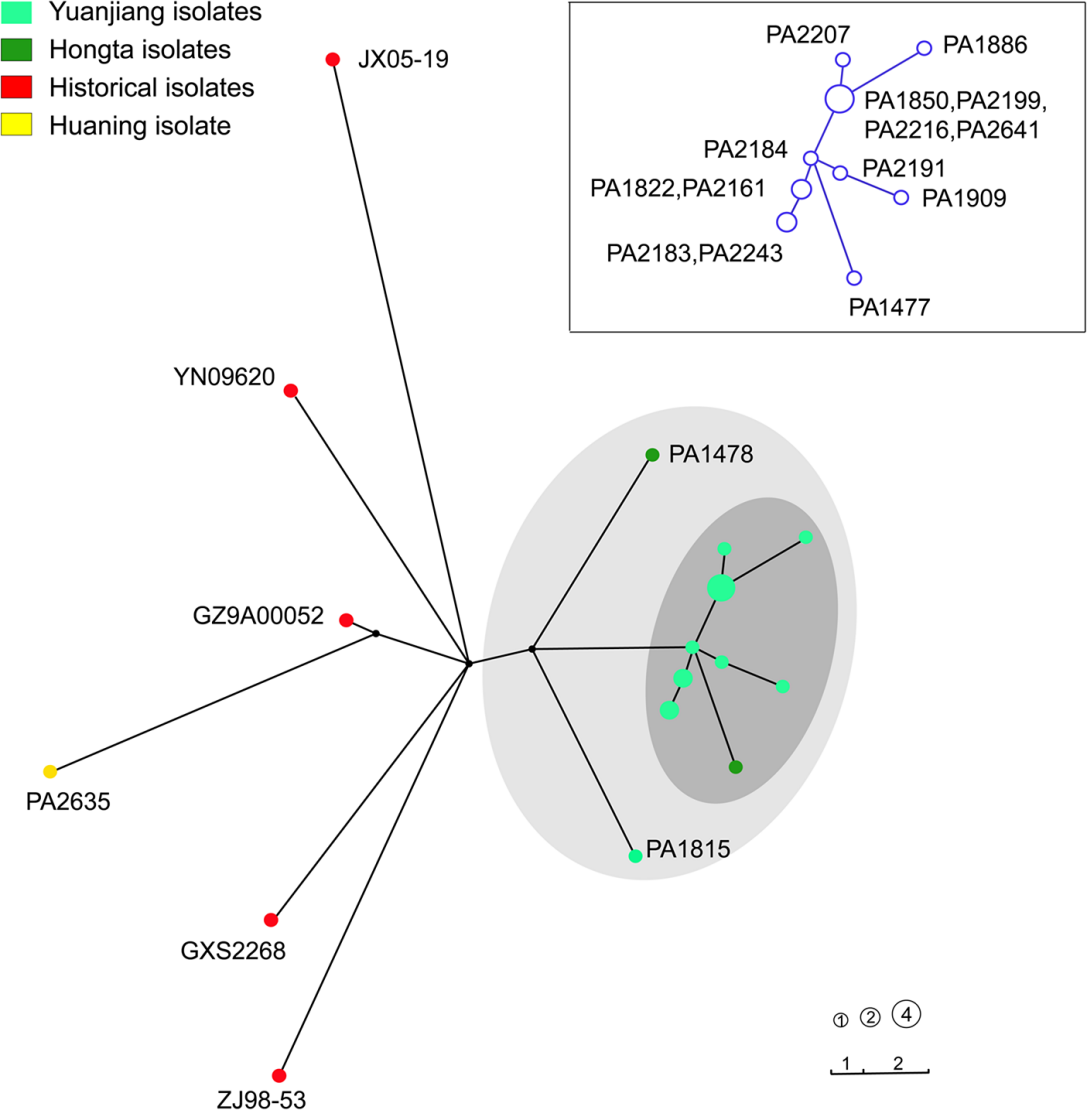
Maximum likelihood tree of 22 *S*. Paratyphi A isolates based on 270 SNPs. In the tree, the sources of the strains are represented by different colors. The types are displayed as circles. The size of each circle represents the number of isolates within this type; the length of each line linking two circles represent the number of SNPs between two types. The isolates possessing common SNP types in the Yuanjiang outbreak are marked with the dark-gray ellipse; one Yuxi isolate is also included. The gray ellipse includes all the Yuanjiang and Yuxi isolates. The isolate codes in the dark-gray ellipse are marked in a copy of these branches in the upper right.

We randomly selected 14 isolates from the Yuanjiang outbreak and 3 epidemic isolates from Hongta district and Huaning county based on PFGE subtyping differences for the whole-genome sequencing. Regarding the phylogeny inferred from the core genome SNPs, 13 of the 14 isolates from Yuanjiang formed one tight clade (Fig 4). This clade showed that a predominant clone circulated in Yuanjiang, though mutations were also observed during transmission among the patients in the outbreak, which was supported by the PFGE analysis (Figs 3 and S3). A distinct strain (PA1815) from Yuanjiang, which may represent a different clone, was also found (Fig 4). Thirteen of the 14 Yuanjiang isolates are closely related to PA1477 from the Hongta district. These 13 Yuanjiang isolates may have been transmitted from Hongta at least once, with a pathogen that is similar to PA1477. Moreover, we observed a close relationship between the epidemic case isolates and the hospital wastewater isolates; no SNP was found between one case isolate and one wastewater isolate. However, one Yuanjiang case isolate (PA1815) differed from the other Yuanjiang outbreak isolates by 14 SNPs. We speculate that this isolate had either been resident in Yuanjiang for a long time or was transmitted by a lineage in Hongta that was not observed by the sequence analysis.

### Intervention and termination of the outbreak

The epidemiological and laboratory investigations suggested roles for both vegetables and hospital wastewater in the outbreak. On October 2nd, 2010, immediately after the detection of *S*. Paratyphi A isolates in the untreated hospital effluent, measures were taken to control the outbreak and to interrupt the transmission of paratyphoid fever in Yuanjiang county. Public health education, additional sampling and the chlorination of municipal water were performed. Additionally, wastewater disinfection and patient waste management control were strengthened in the hospitals. Individual patient waste was sterilized with 20% sodium hypochlorite for approximately 30 minutes prior to the communal collection of waste. Additionally, calcium hypochlorite powder was automatically (final concentration of 20%) added into the pooled sewage from the hospitals and treated for eight hours before discharge. In addition, the disinfected wastewater was cultured for *S*. Paratyphi A and fecal coliform bacteria every week by the local CDC to ensure the effectiveness of the measures. On October 8th, 2010, the vegetable farmers were notified to stop planting on the parcel of land near County People's Hospital, and the selling of raw vegetables to restaurants was prohibited. The number of cases decreased rapidly in the ensuing months (Fig 1).

In March 2011, the number of reported paratyphoid fever cases increased slightly (Fig 1). Four months after the public health measures were relaxed, some restaurants, farmers’ markets and street vendors resumed selling raw vegetables cultivated from the patch near the hospital. In early April 2011, the local government issued a stricter injunction to prohibit the planting of vegetables used for cold dishes and destroyed all the vegetables from the plot near County People’s Hospital. After May, the incidence of enteric fever cases dropped to 11 cases within 7 months, similar to the pre-outbreak incidence baseline.

## Discussion

The Yuanjiang paratyphoid fever outbreak of 2010–2011 is a classic example of a paratyphoid fever outbreak in a developing economy. The outbreak occurred in an area with low sanitary protection, poor wastewater disposal and drought. Though people are the only natural host and reservoir for *S*. Typhi and *S*. Paratyphi A, the two pathogens could survive in variant environments outside of humans, such as water and foods. *S*. Typhi can survive for days in groundwater, pondwater, or seawater, and for months in contaminated eggs and frozen oysters [27–30]. For S. Paratyphi A, it could survive 3 days in river water or polluted water, more than 10 days in contaminated razor calms [31]. Surveillance showed that waterborne causes accounted for 53% of all outbreaks of typhoid and paratyphoid fever during 2004–2007 in China [8]. Raw vegetable-associated enteric fever outbreaks have been reported in other countries [6, 32, 33]. Although *S*. Paratyphi A isolates were not obtained directly from the vegetables examined, the abnormal fecal coliform counts provided indirect evidence of the contamination of the vegetables. When the consumption of these vegetables was prohibited, along with the imposition of strict hospital sewage treatment policies, the number of cases of this disease was radically reduced. Although changes in temperature could not be excluded as a factor in reducing disease during the paratyphoid fever outbreak, we emphasize that a second wave of cases were reported when raw vegetables from the same garden plot were again sold. Indeed, only when this source of vegetables was completely prohibited did the case number drop back to background. Considering the continuation of paratyphoid fever epidemic in the Yuxi region in 2011 and 2012, while the cases in Yuanjiang county decreased to the baseline before the outbreak, it is clear that the comprehensive interventions, largely by the government, played a very important role in interrupting the transmission of paratyphoid fever in developing areas.

In this outbreak, an uncommon but critical role of untreated hospital wastewater was revealed. From January to November 2010, County People's Hospital admitted more than 300 paratyphoid fever patients while the wastewater was not treated appropriately. The outbreak was most likely sustained by the transmission of the pathogen from the hospital to the community. The associations between paratyphoid fever and the consumption of uncooked vegetables obtained from street stalls, the Ximen Farm Product Market and small restaurants indicate that the consumption of raw vegetables, especially dining at a location with poor sanitation may increase the risk of exposure to *S*. Paratyphi A. The vegetables supplied by the local farmers’ market to street vendors and restaurants and subsequently served in cold dishes as seasoning were continually recontaminated from a source where an urban drain mixed with effluent from County Peoples’ Hospital. Our findings highlight the need for healthcare institutions to take preventative action to mitigate outbreaks or epidemics in the populations they serve.

Molecular subtyping combined with genome sequencing and epidemiological studies strongly support a transmission link between an *S*. Paratyphi A clone from a recent Yuxi epidemic and the Yuanjiang outbreak. These data show that when *S*. Paratyphi A is introduced into a population by the use of contaminated ground water for irrigation due to drought, the continued cycle of contaminated water from hospitals combined with the regional habit of eating uncooked vegetables lead to ongoing cases.

*S*. Typhi and *S*. Paratyphi isolates with decreased susceptibility to ciprofloxacin could result in more frequent treatment failure than susceptible strains among patients with typhoid or paratyphoid fever [34–36]. In this study, it is possible that patients infected with *S*. Parayphi A experienced poor response to therapy or treatment failure, because all isolates from the outbreak displayed decreased susceptibility to ciprofloxacin *in vitro*. Therefore, the combinational treatment with fluoroquinolones and cephalosporins was administrated since all isolates were sensitive to ceftriaxone. Unfortunately, the carriage of *S*. Parayphi A was not confirmed in patients before they were discharged and few medical records were obtained simultaneously. The data from this study suggests that it is very important to design treatment procedure based on the bacterial resistant profile to avoid treatment failure and necessary to confirm the carriage of bacterial pathogen before the discharge of patients from hospital.

There were some limitations of this study. First, recall bias of cases and controls could not be avoided, especially the lack of details of the eaten vegetables concerning which vegetables were associated with illness. The other is, we didn’t recover any *S*. Paratyphi A isolates from raw vegetables. Though isolation of *S*. Paratyphi A from environmental water and food is quite difficult, to get *S*. Paratyphi A isolates from vegetables will present powerful support for its spread. However in this study, elimination of risk factors (i.e., raw vegetables) arrested the outbreak of paratyphoid fever will provide evidence for supporting the identification of the risk factor in this outbreak. Another lesson from this outbreak control study is the earlier investigation and following by intervention response will reduce the scale of the outbreak.

Safe water and good hygiene are critical for prevention of fecal-oral transmission of infectious diseases. In this case, lack of well water for irrigation, a result of prevailing drought conditions, played an important role, resulting in the use of wastewater to irrigate vegetable fields. It is necessary to establish separate agricultural irrigation water and waste water systems, and it is also important to enhance the management and treatment of patients, to strengthen the monitoring of medical waste.

## Supporting Information

S1 Checklist STROBE checklist for case control studies. (DOC)

S1 Fig The geographic position of Yuanjiang county on the map of China. (TIF)

S2 Fig Incidence map of paratyphoid fever in the different towns in Yuanjiang during the outbreak in 2010–2011. (TIF)

S3 Fig PFGE patterns of the *S*. Paratyphi A isolates digested with *Xba*I and *Spe*I. A: PFGE patterns of isolates recovered from sources in Yuxi city from 2008–2009. B: PFGE patterns of outbreak strains in Yuanjiang county. The colored slashed box shows the matched patterns between the outbreak and endemic isolates from Yuxi city.
(TIF)

S1 Table *S*. Paratyphi A isolates with whole-genome sequencing used in this study. (DOC)
