# Supplementary material for: A Large-Scale Community-Based Outbreak of Paratyphoid Fever Caused by Hospital-Derived Transmission in Southern China
Source: PLoS Negl Trop Dis. 2015 Jul 17;9(7):e0003859. doi: 10.1371/journal.pntd.0003859 (PMC4506061; doi:10.1371/journal.pntd.0003859)
Supplement: S1 Checklist — (DOC) [file pntd.0003859.s001.doc]

STROBE Statement—Checklist of items that should be included in reports of ***case-control studies***

|  | Item No | Recommendation |
| --- | --- | --- |
| **Title and abstract** | 1 | (*a*) Done. See abstract (page 2). |
|  |  | (*b*) Done. See abstract (page 2). |
| Introduction | | |
| Background/rationale | 2 | Done. See introduction (page 5). |
| Objectives | 3 | Done. See last paragraph of the introduction (page 5-6). |
| Methods | | |
| Study design | 4 | Done. See materials and methods (page 6). |
| Setting | 5 | Done. See materials and methods (page 6-7). |
| Participants | 6 | Done. See materials and methods (page 6). |
| Variables | 7 | Done. See materials and methods (page 6-7). |
| Data sources/ measurement | 8* | Done. See materials and methods (page 6-7). |
| Bias | 9 | Done. See results (page 12). |
| Study size | 10 | Done. See results (page 11). |
| Quantitative variables | 11 | Not done. |
| Statistical methods | 12 | Done. See materials and methods (page 7), results (page11-12) |
| Results | | |
| Participants | 13* | Done. See results (page 11-12). |
| Descriptive data | 14* | Done. See results (page 11-12). |
| Outcome data | 15* | Done. See results (page 11-12). |
| Main results | 16 | Done. See results (page 11-12). |
| Other analyses | 17 | Done. See results (page 11-12). |
| Discussion | | |
| Key results | 18 | Done. See discussion (page 18-19). |
| Limitations | 19 | Done. See discussion (page 20). |
| Interpretation | 20 | Done. See discussion (page 18-20). |
| Generalisability | 21 | Done. See discussion (page 18-20). |
| Other information | | |
| Funding | 22 | The authors appreciate the financial support for Priority Project on Infectious Disease Control and Prevention from the Ministry of Science and Technology, People’s Republic of China. There is no conflict of interest. |

*Give information separately for cases and controls.

**Note:** An Explanation and Elaboration article discusses each checklist item and gives methodological background and published examples of transparent reporting. The STROBE checklist is best used in conjunction with this article (freely available on the Web sites of PLoS Medicine at http://www.plosmedicine.org/, Annals of Internal Medicine at http://www.annals.org/, and Epidemiology at http://www.epidem.com/). Information on the STROBE Initiative is available at http://www.strobe-statement.org.
