# Supplementary figures and images for: A Large-Scale Community-Based Outbreak of Paratyphoid Fever Caused by Hospital-Derived Transmission in Southern China
Source: PLoS Negl Trop Dis. 2015 Jul 17;9(7):e0003859. doi: 10.1371/journal.pntd.0003859 (PMC4506061; doi:10.1371/journal.pntd.0003859)

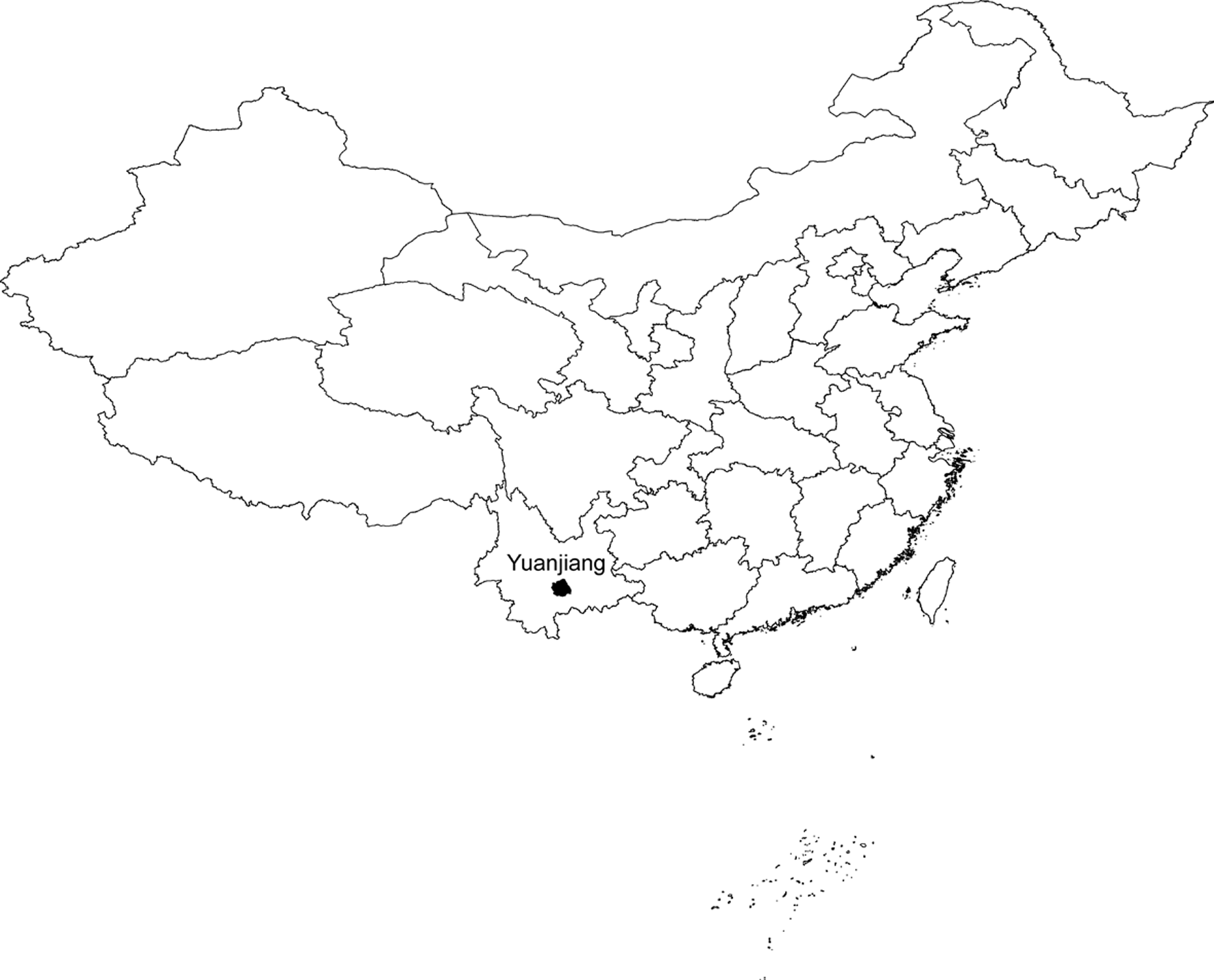

Supplement: S1 Fig — (TIF) [file pntd.0003859.s002.tif]

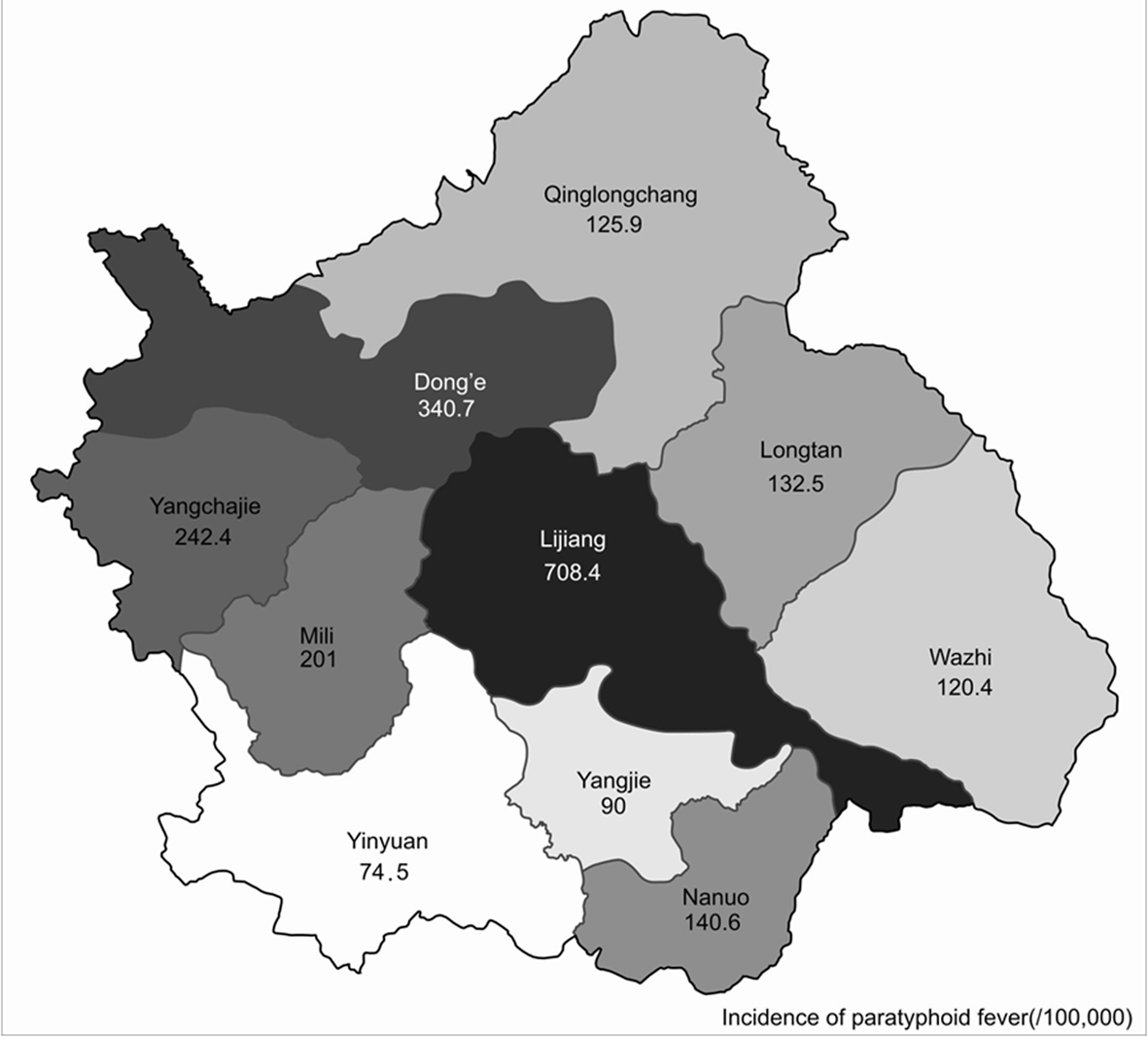

Supplement: S2 Fig — (TIF) [file pntd.0003859.s003.tif]

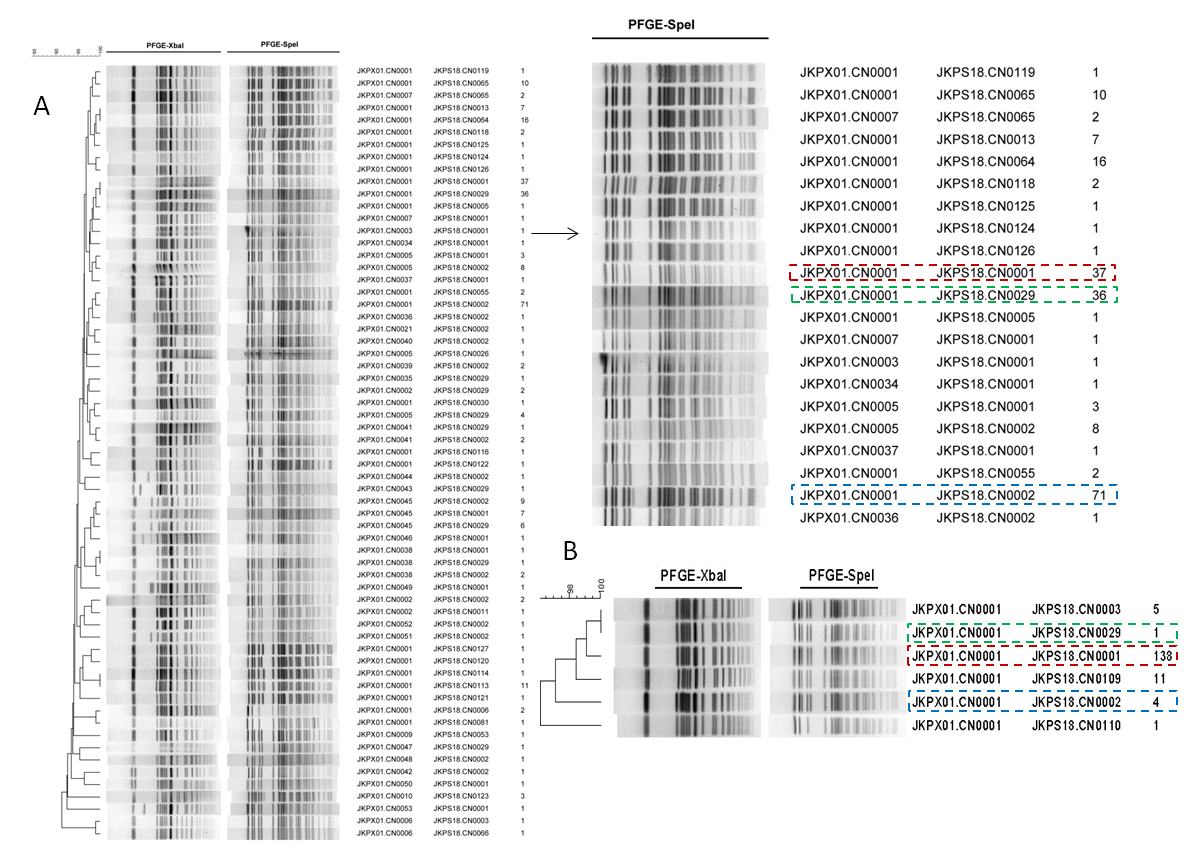

Supplement: S3 Fig — A: PFGE patterns of isolates recovered from sources in Yuxi city from 2008–2009. B: PFGE patterns of outbreak strains in Yuanjiang county. The colored slashed box shows the matched patterns between the outbreak and endemic isolates from Yuxi city. (TIF) [file pntd.0003859.s004.tif]
