# Supplementary material for: A Large-Scale Community-Based Outbreak of Paratyphoid Fever Caused by Hospital-Derived Transmission in Southern China
Source: PLoS Negl Trop Dis. 2015 Jul 17;9(7):e0003859. doi: 10.1371/journal.pntd.0003859 (PMC4506061; doi:10.1371/journal.pntd.0003859)
Supplement: S1 Table — (DOC) [file pntd.0003859.s005.doc]

**S1 Table.** *S.* Paratyphi A isolates with whole-genome sequencing used in this study.

| Strain | Biological information | | | |  | NGS information | | | | | Reference |
| --- | --- | --- | --- | --- | --- | --- | --- | --- | --- | --- | --- |
|  | Isolation date | Source | District | PFGE Pattern (*Xba*I /*Spe*I) |  | No. of Scaffolds | N50 | Total Length | Read Length | No. of Reads |  |
| PA2641 | 2010-2-8 | Patient blood | Yuanjiang | JKPX01.CN0001/ JKPS18.CN0113 |  | 26 | 325,468 | 4,813,373 | 90:90 | 6,582,704 | This study |
| PA1909 | 2010-9-24 | Patient blood | Yuanjiang | JKPX01.CN0001/ JKPS18.CN0001 |  | 27 | 325,590 | 4,952,200 | 90:90 | 6,524,310 | This study |
| PA1886 | 2010-9-30 | Patient blood | Yuanjiang | JKPX01.CN0001/ JKPS18.CN0109 |  | 27 | 325,584 | 4,903,091 | 90:90 | 6,589,928 | This study |
| PA1815 | 2010-10-1 | Patient blood | Yuanjiang | JKPX01.CN0001/ JKPS18.CN0110 |  | 27 | 325,442 | 4,807,154 | 90:90 | 6,645,550 | This study |
| PA1850 | 2010-10-10 | Patient blood | Yuanjiang | JKPX01.CN0001/ JKPS18.CN0029 |  | 31 | 325,588 | 4,809,082 | 90:90 | 6,500,278 | This study |
| PA2243 | 2010-10-11 | Sewage water | Yuanjiang | JKPX01.CN0001/ JKPS18.CN0001 |  | 27 | 325,575 | 4,912,684 | 90:90 | 5,927,486 | This study |
| PA2199 | 2010-10-13 | Patient blood | Yuanjiang | JKPX01.CN0019/ JKPS18.CN0001 |  | 27 | 325,452 | 4,907,104 | 90:90 | 5,947,558 | This study |
| PA1822 | 2010-10-16 | Patient blood | Yuanjiang | JKPX01.CN0001/ JKPS18.CN0003 |  | 27 | 325,588 | 4,808,520 | 90:90 | 6,515,906 | This study |
| PA2161 | 2010-12-9 | Patient blood | Yuanjiang | JKPX01.CN0001/ JKPS18.CN0002 |  | 27 | 325,574 | 4,718,472 | 90:90 | 5,906,580 | This study |
| PA2183 | 2011-1-31 | Patient blood | Yuanjiang | JKPX01.CN0022/ JKPS18.CN0128 |  | 27 | 325,479 | 4,711,344 | 90:90 | 6,489,588 | This study |
| PA2191 | 2011-2-7 | Patient blood | Yuanjiang | JKPX01.CN0018/ JKPS18.CN0001 |  | 27 | 325,580 | 4,734,406 | 90:90 | 5,907,424 | This study |
| PA2184 | 2011-2-10 | Sewage water | Yuanjiang | JKPX01.CN0001 /JKPS18.CN0001 |  | 27 | 325,587 | 4,715,039 | 90:90 | 6,475,162 | This study |
| PA2207 | 2011-3-23 | Patient blood | Yuanjiang | JKPX01.CN0001 /JKPS18.CN0001 |  | 27 | 325,563 | 4,753,411 | 90:90 | 5,764,408 | This study |
| PA2216 | 2011-4-29 | Patient blood | Yuanjiang | JKPX01.CN0018/ JKPS18.CN0002 |  | 27 | 325,633 | 4,753,553 | 90:90 | 5,904,298 | This study |
| PA1477 | 2009-5-6 | Patient blood | Yuanjiang | JKPX01.CN0001 /JKPS18.CN0001 |  | 27 | 325,589 | 4,772,943 | 90:90 | 5,888,172 | This study |
| PA1478 | 2009-5-6 | Patient blood | Yuanjiang | JKPX01.CN0001/ JKPS18.CN0002 |  | 29 | 313,011 | 4,769,162 | 90:90 | 5,958,258 | This study |
| PA2635 | 2010-5-14 | Patient blood | Yuanjiang | JKPX01.CN0001/ JKPS18.CN0065 |  | 28 | 325,563 | 4,809,698 | 90:90 | 5,940,552 | This study |
| GXS2268 | 2007 | Patient blood | Guangxi | JKPX01.CN0001/ JKPS18.CN0026 |  |  |  |  |  |  | [25] |
| GZ9A00052 | 2000 | Patient blood | Guizhou | JKPX01.CN0003/ JKPS18.CN0029 |  |  |  |  |  |  | [25] |
| YN09620 | 2009 | Patient blood | Yunnan | JKPX01.CN0009/ JKPS18.CN0053 |  |  |  |  |  |  | [25] |
| ZJ98-53 | 1998 | Patient blood | Zhejiang | JKPX01.CN0010/ JKPS18.CN0137 |  |  |  |  |  |  | [25] |
| JX05-19 | 2005 | Environment | Jiangxi | JKPX01.CN0026/ JKPS18.CN0062 |  |  |  |  |  |  | [25] |
| ATCC9150 | 1992 | Patient | ND | JKPX01.CN0030/ JKPS18.CN0165 |  |  |  |  |  |  | [26] |
